# Distinct Phenotypic Differences Associated with Differential Amplification of Receptor Tyrosine Kinase Genes at 4q12 in Glioblastoma

**DOI:** 10.1371/journal.pone.0071777

**Published:** 2013-08-21

**Authors:** Anna Burford, Suzanne E. Little, Alexa Jury, Sergey Popov, Ross Laxton, Lawrence Doey, Safa Al-Sarraj, Juliane M. Jürgensmeier, Chris Jones

**Affiliations:** 1 Divisions of Molecular Pathology, The Institute of Cancer Research, Sutton, United Kingdom; 2 Cancer Therapeutics, The Institute of Cancer Research, Sutton, United Kingdom; 3 Neuropathology, King's College Hospital, London, United Kingdom; 4 AstraZeneca, Alderley Park, Macclesfield, United Kingdom; University of Portsmouth, School of Pharmacy & Biomedical Sciences, United Kingdom

## Abstract

Gene amplification at chromosome 4q12 is a common alteration in human high grade gliomas including glioblastoma, a CNS tumour with consistently poor prognosis. This locus harbours the known oncogenes encoding the receptor tyrosine kinases *PDGFRA*, *KIT*, and *VEGFR2*. These receptors are potential targets for novel therapeutic intervention in these diseases, with expression noted in tumour cells and/or associated vasculature. Despite this, a detailed assessment of their relative contributions to different high grade glioma histologies and the underlying heterogeneity within glioblastoma has been lacking. We studied 342 primary high grade gliomas for individual gene amplification using specific FISH probes, as well as receptor expression in the tumour and endothelial cells by immunohistochemistry, and correlated our findings with specific tumour cell morphological types and patterns of vasculature. We identified amplicons which encompassed *PDGFRA* only, *PDGFRA/KIT*, and *PDGFRA/KIT/VEGFR2*, with distinct phenotypic correlates. Within glioblastoma specimens, *PDGFRA* amplification alone was linked to oligodendroglial, small cell and sarcomatous tumour cell morphologies, and rare *MGMT* promoter methylation. A younger age at diagnosis and better clinical outcome in glioblastoma patients is only seen when *PDGFRA* and *KIT* are co-amplified. *IDH1* mutation was only found when all three genes are amplified; this is a subgroup which also harbours extensive *MGMT* promoter methylation. Whilst *PDGFRA* amplification was tightly linked to tumour expression of the receptor, this was not the case for *KIT* or *VEGFR2.* Thus we have identified differential patterns of gene amplification and expression of RTKs at the 4q12 locus to be associated with specific phenotypes which may reflect their distinct underlying mechanisms.

## Introduction

Molecular profiling of glioblastoma has recently uncovered a catalogue of DNA copy number changes [1], [2], gene expression [1], [3], [4] and DNA methylation patterns, and mutations [1], [2] which appear to drive subgroups of the disease. Amongst this data, gene amplification at chromosome 4q12 has been reported in 74/463 (16%) cases assessed by array-based comparative genomic hybridisation (aCGH) [5], and is linked to a subclass of tumours associated with a younger age at diagnosis, Proneural gene expression signature, *IDH1* mutation, and a CpG island methylator phenotype [4], [6].

Within the 4q12 amplicon reside genes encoding the receptor tyrosine kinases (RTKs) and drug targets *PDGFRA*, *KIT*, and *KDR (VEGFR2)*. Bulk profiling of tumour specimens suggests that only PDGFRA is common to 4q12 amplified cases, with 39% of these harbouring *PDGFRA* amplification alone, 23% including *PDGFRA* and *KIT*, and 38% encompassing all three RTKs [5]. Recent studies using multi-colour fluorescent *in situ* hybridisation (FISH) on archival pathology specimens has demonstrated the marked genetic heterogeneity of glioblastoma specimens in relation to RTK gene amplification [5], [7], [8]. Individual cells, or small foci of cells may harbour increased DNA copy number of a gene that is not found amplified in the majority of cells, and would not be detected by bulk tumour aCGH [7]. Thus there may be instances in which isolated cells/foci may harbour *KIT* and/or *VEGFR2* amplification in the absence of copy number gain of *PDGFRA*, as has been implied by previous FISH studies of all three genes separately [9].

The specific RTKs involved at 4q12 have translational interest for several reasons. Receptor expression of PDGFRA and KIT has been found in numerous malignancies, particularly sarcomas such as GIST (gastro-intestinal stromal tumour), against which small molecule inhibitors have demonstrated clinical response [10], [11]. In fact selectivity between these class III RTKs can be difficult to achieve, so many of these targeted inhibitors act on both receptors [12]–[14]. In glioblastoma, amplification/overexpression of PDGFRA has provided the rationale for clinical studies, with disappointing results to date [15]–[17]. The role of KIT expression in glioblastoma, and thus the necessity of targeting the receptor clinically, is less clear.

By contrast, VEGFR2 does not tend to be expressed in cancer cells, but is extensively expressed within the tumour endothelium, and is the major transducer of VEGF signals, resulting in increased angiogenesis through endothelial cell survival, proliferation and migration [18], [19]. The critical role of VEGF/VEGFR2 in tumour angiogenesis has been demonstrated in both animal studies and clinical trials in numerous human malignancies, including glioblastoma [20]–[22]. PDGFRA and KIT too, may be expressed in the glioblastoma-associated vasculature, although they have been less extensively studied. Consistent with a potential role in angiogenesis, KIT is found on circulating endothelial precursor cells and human umbilical vein endothelial cells (HUVEC) [23], [24], and its ligand stem cell factor (SCF) has been implicated as both an autocrine and paracrine growth factor for gliomas. In comparison, the role of PDGFRA has been much less appreciated [25], [26], although there is evidence to suggest that signalling through the receptor plays an important role in regulating VEGF-driven tumour angiogenesis particularly when tumour cells are deficient in VEGF production.

The aim of this study was to assess the relative contributions of PDGFRA, KIT and VEGFR2 to high grade glioma tumour biology, in terms of gene amplification and expression in the tumour cells and associated vasculature. Given the heterogeneity of clinical glioblastoma specimens, we further correlated these data with tumour cell morphological subtypes, specific patterns of vascular proliferation and known genetic prognostic markers such as IDH1 mutation [27] and MGMT promoter methylation [28]. We identified the unique observation that specific patterns of gene amplification at 4q12 were associated with distinct phenotypes in glioblastoma specimens.

## Materials and Methods

### Patient samples

A series of 362 formalin-fixed, paraffin-embedded (FFPE) samples were retrieved after approval from Wandsworth Research Ethics Committee from 342 consecutive patients diagnosed with glioblastoma multiforme (WHO grade IV, n = 276), anaplastic astrocytoma (WHO grade III, n = 17) or anaplastic oligodendroglioma (WHO grade III, n = 49) within the last 5 years from the archives of King's College Hospital, London, with diagnosis confirmed by re-review (SP, SA-S). All the samples enrolled in the present study were unlinked and unidentified from their donors. Due the retrospective nature of the study, no written informed consent from patients was obtained, with the exception of the UK samples obtained after 2006, where all patients signed a written informed consent, following the UK Human Tissue Act approved in that year. Other grade III/IV glioma subtypes such as mixed oligoastrocytomas were not included in the present study. The age range of the patients was 26–83 years (median 58 years), and comprised 61% males to 39% females. Clinical follow-up was available for 329 patients, with a median survival of 6.5 months (range 2 days –5.6 years).

A detailed morphological assessment of the 276 GBM samples was also carried out and the predominant cell types recorded as either as fibrillary (composed of pleomorphic cells with scant or more prominent pink cytoplasm; some cells can demonstrate cytoplasmic extensions); gemistocytic (>20% cells having abundant, glassy cytoplasm, eccentric nuclei); giant cell (composed primarily of bizarre, pleomorphic, often multinucleated cells); small cell (monomorphic round to oval nuclei, uniform in size with bland chromatin but with high mitotic activity; occasional clear perinuclear haloes, branching capillaries and microcalcifications can be seen); oligodendroglial (cytologic monotony with rounded, bland nuclei surrounded by perinuclear haloes; in some cases branching, “chicken wire”-like capillaries); or sarcomatous (consisting of fascicles of spindle cells, sometimes with collagen depositions; stellate cells in a myxoid stroma can be seen in some cases).

### Tissue microarray construction

1 mm diameter cores from each case were taken and constructed in four TMAs containing approximately 90–100 samples each. In addition, a series of control tissues were included, comprising normal human brain, normal mouse brain, placenta, colon and lung. A further H&E was taken of each TMA and re-reviewed to ensure the presence of tumour cells.

### Immunohistochemistry

Each TMA was serially sectioned at 5 µm and immunohistochemistry was performed. Slides were dewaxed in xylene and rehydrated through a descending ethanol series. The rabbit EnVision™+ HRP System, (DAKO, Carpinteria, CA, USA) was used for PDGFRA (D1E1E) and VEGFR2 (55B11) (Cell Signalling, MA, USA). Antigen retrieval was performed by boiling the slides for 15 min in 10 mM citrate buffer pH6 in a coplin jar and cooled on the bench for 20 min, or for 30 min in DAKO Target Retrieval Solution pH9, respectively. Each section was blocked with DAKO Protein block for 10 min at room temp and incubated with primary antibody for 1 hour at room temp (PDGFRA 1∶100) or overnight at 4°C (VEGFR2 1∶100). The Universal R.T.U. Vectastain Elite ABC Kit (Vector Laboratories, Burlingame, CA, USA) was used for CD117/c-kit (A4502) (DAKO). After blocking, each section was incubated with primary antibody for 1 hour at room temp (1∶250). Staining was completed with a 5 minute incubation with DAKO 3,3′-diaminobenzidine (DAB)+ substrate-chromogen and counterstained with Mayer's Haematoxylin (Sigma-Aldrich, Poole, UK).

### Fluorescence in situ hybridisation

**Figure 1:**
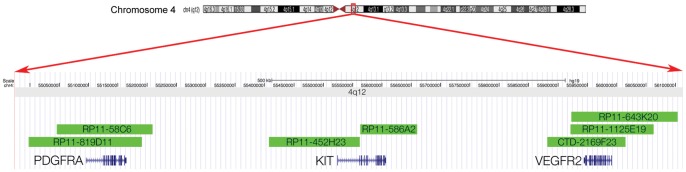
Gene-specific BAC probes used for FISH. Probes (green bars) were selected according the hg19 (February 2009) build of the human genome to selectively overlap with the coding sequence for either PDGFRA (RP11-819D11, RP11-58C6), KIT (RP11-452H23, RP11-586A2) or VEGFR2 (CTD-2169F23, RP11-1125E19, RP11-643K20) alone.

FISH analysis was carried out on FFPE sections as previously described [7]. Briefly, BAC clones were purchased from BACPAC Resources Center (Children's Hospital Oakland Research Institute, Oakland, CA, USA) and FISH-mapped onto metaphase slides to ensure specificity. BAC clones used were: *PDGFRA* – RP11-58C6 and RP11-819D11; KIT – RP11-452H23 and RP11-586A2; VEGFR2– CTD2169F23, RP11-1125E19 and RP11-643K20; centromere 4– RP11-317G22 and RP11-191S2 (Figure 1). BAC DNA was amplified from 10ng starting material using the Illustra^TM^ GenomiPhi^TM^ V2 DNA amplification kit (GE Healthcare, Little Chalfont, UK) according to the manufacturer's instructions. Probes were labelled with either biotin or DIG using the BioPrime^®^ DNA Labeling System (Invitrogen, Paisley, UK). TMAs were stripped of paraffin using xylene, pretreated with 0.2M HCL and 8% sodium thiocyanate at 80°C for 30 minutes, digested in 0.025% pepsin (sigma) in 0.01M HCL at 37°C for 20 minutes before adding a prepared probe mix with (75%) hybridization mix under a coverslip and denatured for 5 minutes at 73°C on a heatblock. They were then incubated overnight in a humidified chamber at 37°C, mounted in Vectashield with DAPI (Vector Laboratories, Peterborough, UK), and captured on the Ariol System (Leica Microsystems, Wetzlar, Germany) using filters for DAPI, Cy3 and FITC. Hybridization was carried out using differential labeling of one gene and one centromeric probe (PDGFRA-Cy3/Cent4-FITC, KIT/Cent4-FITC, VEGFR2-Cy3/Cent4-FITC). Each core was screened for cells with 10 or more gene copies and with a ratio of gene:centromere of 10∶2. Any core with at least one such cell was considered amplified. At least 50 cells per core were screened. All reactions were also assessed on normal brain control cores.

### Genetic analysis

DNA was extracted from paraffin-embedded formalin-fixed tissue using the QIAamp DNA Micro Kit (Qiagen, Crawley, UK). Primers for the 129bp *IDH1* fragment were designed and PCR carried out according to the method in Hartmann *et al.* [29]. The PCR product was sequenced and the *IDH1^R132H^* mutation analysed using Mutation Surveyor (SoftGenetics, Pennsylvania, USA) and manually with 4Peaks (Mekentosj, Aalsmeer, The Netherlands). *MGMT* promoter methylation was assessed by bisulfite conversion of the FFPE-extracted DNA using the Qiagen EpiTect Bisulfite Kit and Methylation-Specific PCR (MSP) using standard primers as previously described [30].

### Statistical analysis

All statistical tests were performed in R2.11.0 (www.r-project.org). Correlations between categorical variables were analysed by Fishers exact test, and between continuous variable by Students t-test. Cumulative survival probabilities were calculated using the Kaplan-Meier method with differences between survival rates analysed with the log-rank test. Important prognostic information (including Karnofsky performance score) was not available for all cases in this retrospective study, so multivariate analysis was not able to be performed. All tests were two-tailed, with a confidence interval of 95%. P values of less than 0.05 were considered statistically significant.

## Results

### Amplification of receptor tyrosine kinase genes at 4q12 confers a poor prognosis in glioblastoma

We used specific FISH probes independently designed to recognise *PDGFRA*, *KIT* and *VEGFR2* genes (Figure 1) to determine the relative frequencies of gene amplification at chromosome 4q12 in our high grade glioma series. Taken together, amplification of any gene or combination of genes at this locus was found in 63/283 (22.2%) cases, including 6/37 (16.2%) anaplastic oligodendroglioma, 3/13 (23.1%) anaplastic astrocytoma, and 54/233 (23.2%) glioblastoma. Although not statistically significant, there appears to be a trend towards the presence of the amplicon being associated with poor clinical outcome in glioblastoma (p = 0.054, log-rank test, overall survival, data not shown).

Taken individually, we observed *PDGFRA* amplification in 56/236 (23.7%) cases (6/37, 16.2% anaplastic oligodendroglioma; 2/11, 18.1% anaplastic astrocytoma; 48/188, 25.5% glioblastoma); *KIT* amplification in 49/235 (20.9%) cases (4/30, 13.3% anaplastic oligodendroglioma; 3/11, 27.3% anaplastic astrocytoma; 42/194, 21.6% glioblastoma); and *VEGFR2* amplification in 26/190 (13.7%) cases (2/27, 7.4% anaplastic oligodendroglioma; 1/9, 11.1% anaplastic astrocytoma; 23/154, 14.9% glioblastoma).

**Figure 2:**
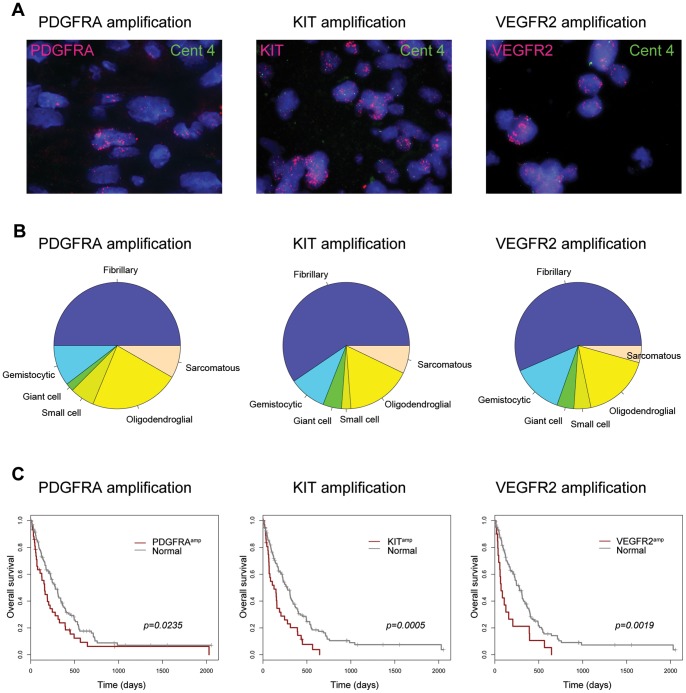
Amplification of 4q12 genes in glioblastoma. (A) Representative FISH images showing individual amplification of *PDGFRA*, *KIT*, and *VEGFR2* in glioblastoma samples RMH6862, RMH6427 and RMH6881, respectively. Original magnification ×1000. (B) Pie charts showing the relative proportions of the predominant cellular morphology in glioblastoma samples for cases with *PDGFRA*, *KIT*, and *VEGFR2* amplification. Fibrillary, purple; gemistocytic, blue; giant cell, green; small cell, light green; oligodendroglial, yellow; and sarcomatous, orange. (C) Kaplan-Meier curves for the effect of amplification of *PDGFRA*, *KIT*, and *VEGFR2* on overall survival in glioblastoma patients. P values are given using the log-rank test for overall survival.

We next assessed these markers specifically in glioblastoma specimens subclassified according to their predominant morphological features (representative FISH images showing amplification of each gene are shown in Figure 2A). Although there is a trend towards *PDGFRA* amplification being more common in three of the six morphological subgroups of glioblastoma, these numbers do not reach statistical significance (predominantly small cell (3/13, 23.1%, p = 0.086, Fishers exact test), oligodendroglial (11/43, 25.6%, p = 0.061, Fishers exact test), and sarcomatous (4/13, 30.8%, p = 0.116, Fishers exact test) morphology (Figure 2B)) compared to *KIT*-amplified (1/15 small cell, 6.7%; 7/40 oligodendroglial, 17.5%; 4/17 sarcomatous, 23.5%) or *VEGFR2*-amplified (1/9 small cell, 11.1%; 4/37 oligodendroglial, 10.8%; 1/10 sarcomatous, 10.0%) cases. All three genes were individually associated with poor clinical outcome when amplified (Figure 2C) (p = 0.0235, p = 0.0005 and p = 0.0019, respectively, log-rank test, overall survival).

### Independent 4q12 gene amplification patterns have phenotypic correlates

**Figure 3:**
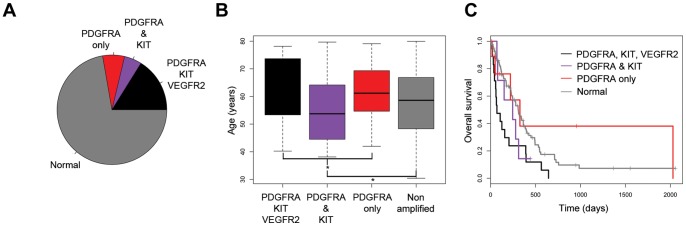
Differential patterns of 4q12 gene amplification. (A) Pie chart showing the relative proportions of distinct gene amplification subtypes in glioblastoma. All three genes, black; *PDGFRA* and *KIT*, purple; *PDGFRA*-only, red; normal copy number of all three genes, grey. (B) Boxplot showing the age distribution of the gene amplification subtypes. * p<0.05, t test. (C) Kaplan-Meier curves for the effect of gene amplification subtypes on clinical outcome.

We next examined the distinct patterns of amplification we observed for each of the three 4q12 genes in our cohort. Where assessable in the same sample, we observed amplification of all three genes in 24/154 (15.6%) glioblastoma cases (24/41, 58.5% of 4q12-amplified cases), *PDGFRA* and *KIT* in 8/154 (5.2%) (8/41, 19.5% 4q12-amplified) and *PDGFRA* only in 9/154 (5.8%) (9/41, 21.9% 4q12-amplified), (Figure 3A). We did not observe *KIT* and/or *VEGFR2* amplification in the absence of PDGFRA amplification in any of our cases.

Tumours with different patterns of 4q12 gene amplification appeared to mark distinct clinicopathological subgroups of glioblastoma. Cases with both *PDGFRA* and *KIT* amplification had a younger age of diagnosis (median = 53.7 yrs) compared with other amplified tumours (median = 60.3 yrs, p = 0.046, t test) (Figure 3B). This is despite the relative clinical outcomes, with tumours amplified for two or three genes doing significantly worse than those amplified for *PDGFRA* alone or harbouring normal copy number (p = 0.0159, log-rank test) (Figure 3C).

Assessable grade III tumours all were found to have all three genes amplified, as was the single case of glioblastoma with *IDH1* mutation (Table 1). MGMT promoter methylation was significantly less frequent in tumours with all three genes amplified (6/19, 31.6%) than the cohort as a whole (154/247, 62.3%, p = 0.026, Fishers exact test); by contrast tumours amplified for *PDGFRA* alone had significantly more *MGMT* promoter methylation (7/9, 77.8%, p = 0.042, Fishers exact test). Those tumours with concurrent *PDGFRA* and *KIT* amplification fell in between these figures (3/7, 42.9%) (Table 1).

**Table 1:** Table of cases for which FISH results were available for all three genes.

RMH_No	PDGFRA_FISH	PDGFRA_IHC	KIT_FISH	KIT_IHC	VEGFR2_FISH	VEGFR2_IHC	Diagnosis	Age_years	IDH1	MGMT
RMH6668	Amplification	Positive	Amplification	Positive	Amplification	Positive	Glioblastoma multiforme	55.96	Negative	**UN**methylated
RMH5696	Amplification	Positive	Amplification	Positive	Amplification	Positive	Glioblastoma multiforme	57.1	Negative	Methylated
RMH6881	Amplification	Positive	Amplification	Positive	Amplification	Negative	Glioblastoma multiforme	44.6	IDH1	Methylated
RMH6357	Amplification	Positive	Amplification	Positive	Amplification	Negative	Glioblastoma multiforme	59.71	Negative	NA
RMH6010	Amplification	Positive	Amplification	Positive	Amplification	Negative	Glioblastoma multiforme	62.88	Negative	NA
RMH6862	Amplification	Positive	Amplification	Positive	Amplification	Negative	Glioblastoma multiforme	67.45	Negative	Methylated
RMH5697	Amplification	Positive	Amplification	Positive	Amplification	Negative	Glioblastoma multiforme	75.96	Negative	**UN**methylated
RMH5685	Amplification	Positive	Amplification	Positive	Amplification	Negative	Glioblastoma multiforme	76.49	Negative	Methylated
RMH6674	Amplification	Positive	Amplification	Positive	Amplification	Negative	Glioblastoma multiforme	78.13	Negative	**UN**methylated
RMH6440	Amplification	Positive	Amplification	Negative	Amplification	Negative	Anaplastic oligodendroglioma	40.24	IDH1	Methylated
RMH6400	Amplification	Positive	Amplification	Negative	Amplification	Negative	Glioblastoma multiforme	49.87	Negative	Methylated
RMH5724	Amplification	Positive	Amplification	Negative	Amplification	Negative	Glioblastoma multiforme	53.34	Negative	**UN**methylated
RMH6417	Amplification	Positive	Amplification	Negative	Amplification	Negative	Glioblastoma multiforme	55.79	Negative	**UN**methylated
RMH6388	Amplification	Positive	Amplification	Negative	Amplification	Negative	Glioblastoma multiforme	55.91	Negative	**UN**methylated
RMH6382	Amplification	Positive	Amplification	Negative	Amplification	Negative	Glioblastoma multiforme	61.47	Negative	**UN**methylated
RMH6433	Amplification	Positive	Amplification	Negative	Amplification	Negative	Glioblastoma multiforme	64.95	Negative	**UN**methylated
RMH5989	Amplification	Positive	Amplification	Negative	Amplification	Negative	Glioblastoma multiforme	69.18	Negative	**UN**methylated
RMH6416	Amplification	Positive	Amplification	Negative	Amplification	Negative	Glioblastoma multiforme	73.68	Negative	Methylated
RMH5981	Amplification	Positive	Amplification	Negative	Amplification	Negative	Anaplastic astrocytoma	75.25	Negative	**UN**methylated
RMH6892	Amplification	Positive	Amplification	Negative	Amplification	Negative	Glioblastoma multiforme	76.04	Negative	**UN**methylated
RMH6926	Amplification	Positive	Amplification	Negative	Amplification	Negative	Glioblastoma multiforme	76.07	Negative	**UN**methylated
RMH6667	Amplification	Positive	Amplification	NA	Amplification	Negative	Glioblastoma multiforme	55.98	Negative	**UN**methylated
RMH6872	Amplification	Negative	Amplification	Positive	Amplification	Negative	Anaplastic oligodendroglioma	50.17	Negative	**UN**methylated
RMH6889	Amplification	NA	Amplification	Negative	Amplification	Negative	Glioblastoma multiforme	51.83	Negative	**UN**methylated
RMH6927	Amplification	Positive	Amplification	Positive	Normal	Negative	Glioblastoma multiforme	66.83	Negative	Methylated
RMH6006	Amplification	Positive	Amplification	Negative	Normal	Negative	Glioblastoma multiforme	38.15	Negative	Methylated
RMH6905	Amplification	Positive	Amplification	Negative	Normal	Negative	Glioblastoma multiforme	43.68	Negative	**UN**methylated
RMH6935	Amplification	Positive	Amplification	Negative	Normal	Negative	Glioblastoma multiforme	45.36	Negative	Methylated
RMH6669	Amplification	Positive	Amplification	Negative	Normal	Negative	Glioblastoma multiforme	53	Negative	**UN**methylated
RMH5726	Amplification	Positive	Amplification	Negative	Normal	Negative	Glioblastoma multiforme	61.49	Negative	**UN**methylated
RMH5966	Amplification	Positive	Amplification	Negative	Normal	Negative	Glioblastoma multiforme	79.65	Negative	NA
RMH5725	Amplification	Negative	Amplification	Negative	Normal	Negative	Glioblastoma multiforme	54.47	Negative	Methylated
RMH6873	Amplification	Positive	Normal	Positive	Normal	Negative	Glioblastoma multiforme	65.5	Negative	Methylated
RMH5684	Amplification	Positive	Normal	Negative	Normal	Positive	Glioblastoma multiforme	41.97	Negative	Methylated
RMH6645	Amplification	Positive	Normal	Negative	Normal	Negative	Glioblastoma multiforme	52.15	Negative	Methylated
RMH5701	Amplification	Positive	Normal	Negative	Normal	Negative	Glioblastoma multiforme	54.66	Negative	**UN**methylated
RMH6887	Amplification	Positive	Normal	Negative	Normal	Negative	Glioblastoma multiforme	61.1	Negative	**UN**methylated
RMH6374	Amplification	Positive	Normal	Negative	Normal	Negative	Glioblastoma multiforme	72.35	Negative	NA
RMH5714	Amplification	Negative	Normal	Negative	Normal	Negative	Glioblastoma multiforme	61.25	Negative	Methylated
RMH5716	Amplification	Negative	Normal	Negative	Normal	Negative	Glioblastoma multiforme	79.08	Negative	Methylated
RMH6925	Amplification	NA	Normal	Negative	Normal	NA	Glioblastoma multiforme	54.91	Negative	Methylated

DNA copy number status, tumour receptor expression, diagnosis, age, IDH1 mutation status and MGMT methylation status are provided. Grey boxes, protein expression in conjunction with gene amplification for a given receptor. NA  =  not available.

### Differential receptor expression and DNA copy number

**Figure 4:**
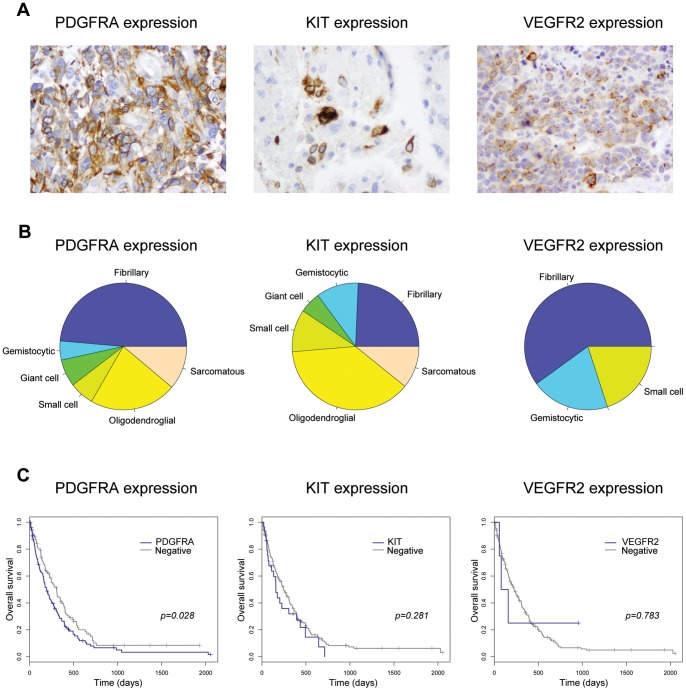
Expression of PDGFRA, KIT and VEGFR2 receptors in glioblastoma tumour cells. (A) Representative images showing tumour cell positivity for PDGFRA, KIT, and VEGFR2 in glioblastoma samples RMH6374, RMH6357 and RMH6691, respectively. Original magnification ×400. (B) Pie charts showing the relative proportions of the predominant cellular morphology in glioblastoma samples for cases overexpressing *PDGFRA*, *KIT*, and *VEGFR2*. Fibrillary, purple; gemistocytic, blue; giant cell, green; small cell, light green; oligodendroglial, yellow; and sarcomatous, orange. (C) Kaplan-Meier curves for the effect of overexpression of *PDGFRA*, *KIT*, and *VEGFR2* on overall survival in glioblastoma patients. P values are given using the log-rank test for overall survival.

In addition to mapping the different DNA copy number patterns of 4q12-amplified genes, we also investigated the protein expression of the receptors themselves in the tumour cells (Figure 4A). PDGFRA was expressed in 196/341 (57.5%) cases (41/49, 83.7% anaplastic oligodendroglioma; 11/19, 57.9% anaplastic astrocytoma; 144/273, 52.7% glioblastoma); KIT expressed in 56/332 (16.9%) cases (17/44, 38.6% anaplastic oligodendroglioma; 2/16, 12.5% anaplastic astrocytoma; 37/272, 13.6% glioblastoma); and VEGFR2 expressed in 5/341 (1.5%) cases (0/48, 0% anaplastic oligodendroglioma; 0/16, 0% anaplastic astrocytoma; 5/257, 1.9% glioblastoma). KIT expression was often restricted to isolated tumour cells, whilst VEGFR2 frequently gave a granulated cytoplasmic staining in addition to decoration of the cell membranes.

As well as distinct patterns of protein expression (Table 1), expression of PDGFRA is associated with gene amplification, with 46/52 (88.5%) amplified cases showing receptor expression, compared to 90/169 (53.3%) normal copy cases (p<0.0001, Fishers exact test). There was also a significant correlation between *KIT* amplification and protein expression, but only in cases where all three genes were amplified (11/25, 44% *KIT*-amplified, 14/140, 10% normal copy number, p = 0.0001, Fishers exact test) and not where *KIT* was amplified with only *PDGFRA* in the absence of *VEGFR2* (1/8, 12.5%, p = 0.396, Fishers exact test). Tumour expression of VEGFR2 was only observed in 2/23 (8.7%) cases with corresponding gene amplification.

Correlating with these differences where phenotypic distinctions associated with tumour cell expression of the three receptors. PDGFRA positive glioblastoma cases showed a trend towards *IDH1* mutation (7/8, 87.5% IDH1 mutant tumours positive for PDGFRA *vs* 135/263, 51.3% *IDH1* wild-type, p = 0.068, Fishers exact test), and exhibited a similar distribution of predominant glioblastoma morphologies as for *PDGFRA* gene amplification (Figure 4B). KIT receptor overexpressing tumours showed a rather different spectrum of morphological correlates, with expression significantly enriched in oligodendroglial tumours (14/37, 37.8% KIT positive cases oligodendroglial *vs* 50/235 negative, 21.3%, p = 0.0136, Fishers exact test). Intriguingly, KIT receptor expression was also associated with an increased incidence of *MGMT* promoter methylation (19/32, 59.4% *vs* 34/268, 12.7%, p = 0.011, log-rank test). The few cases found to express the VEGFR2 receptor in the tumour cells precluded robust phenotypic conclusions being drawn. Of note, only PDGFRA overexpression conferred a worse overall survival in glioblastoma patients (p = 0.028, log-rank test) (Figures 4C).

### Loss of vascular receptor expression is a negative prognostic indicator in glioblastoma

**Figure 5:**
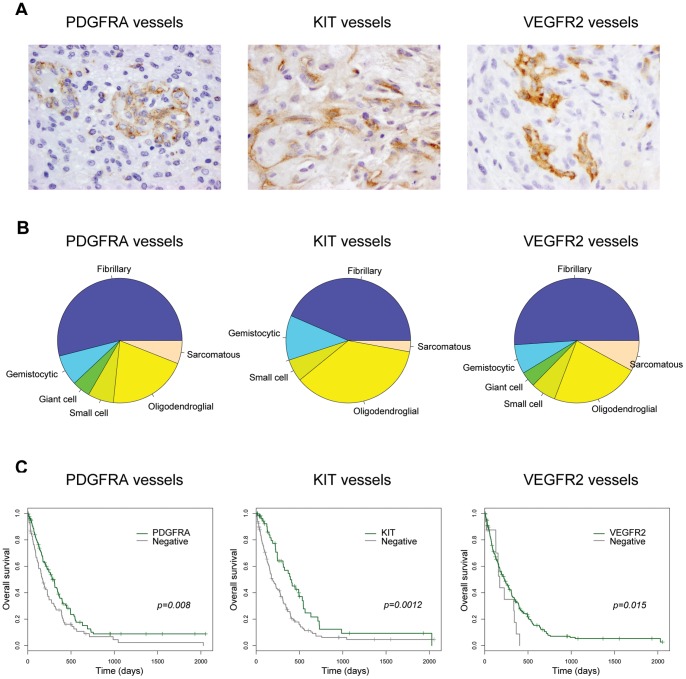
Vascular endothelial cell expression of PDGFRA, KIT and VEGFR2 receptors in glioblastoma. (A) Representative images showing endothelial cell expression of PDGFRA, KIT, and VEGFR2 in glioblastoma samples RMH6401, RMH6657 and RMH6713, respectively. Original magnification ×400. (B) Pie charts showing the relative proportions of the predominant cellular morphology in glioblastoma samples for cases with vessels expressing *PDGFRA*, *KIT*, and *VEGFR2*. Fibrillary, purple; gemistocytic, blue; giant cell, green; small cell, light green; oligodendroglial, yellow; and sarcomatous, orange. (C) Kaplan-Meier curves for the effect of vascular expression of *PDGFRA*, *KIT*, and *VEGFR2* on overall survival in glioblastoma patients. P values are given using the log-rank test for overall survival.

As all three RTKs have been implicated in glioblastoma angiogenesis, finally we also investigated their expression in the tumour vasculature (Figure 5A). PDGFRA positive vessels were found in 170/324 (52.5%) cases (15/44, 34.1% anaplastic oligodendroglioma; 5/18, 27.8% anaplastic astrocytoma; 150/262, 57.3% glioblastoma); KIT positive vessels in 79/302 (26.2%) cases (8/41, 19.5% anaplastic oligodendroglioma; 2/15, 13.3% anaplastic astrocytoma; 69/246, 28.0% glioblastoma); and VEGFR2 positive vessels in 306/341 (89.7%) cases (41/49, 83.7% anaplastic oligodendroglioma; 12/17, 70.6% anaplastic astrocytoma; 253/275, 92% glioblastoma). There were no correlations of endothelial cell positivity and gene amplification or tumour cell expression for any of the receptors (Table S1).

There were few tumour phenotypic differences between receptor-positive and negative vessels, save for an increased proportion of oligodendroglial-predominant glioblastoma specimens with *KIT* positive endothelial cells (25/69, 36.2% *vs* 33/177, 18.6%, p = 0.0022, Fishers exact test) (Figure 5B). Despite the wide variation in vascular expression of the receptors (28–92%), for all three RTKs, a positive tumour vasculature conferred a better overall survival compared with cases whose vessels were negative (p = 0.008 PDGFRA, p = 0.0012 KIT, p = 0.015 VEGFR2, log-rank test) (Figure 5C). VEGFR2-negative vessels were uncommon (8% glioblastoma cases), however were found to have less glomeruloid proliferation (9/20, 45% *vs* 188/254. 74%, p = 0.00692, Fishers exact test), and a total absence of arcade-like structures (0/20, 0% *vs* 57/254, 22.4%, p = 0.0077, Fishers exact test).

## Discussion

Recently we and others have established that the profound intratumoural heterogeneity of glioblastomas may extend to the molecular level in terms of gene amplification [5], [7], [8]. There appear to exist subclones within individual tumour specimens which harbour distinct oncogenic gene amplifications which may be mutually exclusive with each other [5], [7], [8]. For this reason we have chosen in the present study to use FISH to assess 4q12 gene amplification in glioblastoma and other high grade gliomas in order not to exclude any samples with only rare events that may be missed by bulk tumour profiling.

As we include some cases with only one cell with *bona fide* amplification in our tissue microarray screen, we have identified a higher frequency of 4q12-amplified cases than in previous reports [5], [31], although these data are not directly comparable as the microarray- or PCR-based methods used bulk tumour profiling. In our hands, amplification of any of *PDGFRA*, *KIT* or *VEGFR2* is found in nearly a quarter of glioblastoma cases, with individual genes ranging from 14–25%, which may itself be an underestimate due to sampling. The mechanistic significance of isolated tumour cells harbouring distinct gene amplifications in a wider bulk population without these events is still unclear, although *in vitro* these subpopulations are dynamic under ligand or receptor inhibitor exposure [5], and may contribute to the clinical failure of single targeted inhibitors.

*PDGFRA* amplification has been linked with receptor overexpression, younger age at diagnosis, *IDH1* mutation, better clinical outcome and oligodendroglial morphology [4], [6]. In this study, the link of DNA copy number to protein expression holds regardless of amplification pattern at 4q12, and the morphological correlates can also be extended to include small cell and sarcomatous tumour cell histologies. A younger age at diagnosis and better clinical outcome is only seen when *PDGFRA* and *KIT* are co-amplified. 4q12 amplification has been linked to a Proneural gene expression profile and IDH1 mutation [4], [6]. The association with *IDH1* mutation in our series is only significant when all three genes are amplified. Given the relatively poor prognosis of 4q12 amplified cases in comparison with the *IDH1*/Proneural tumours, it is possible that these cases represent a distinct clinicopathological subgroup of these GBM. Of note, this is a subgroup which also harbours considerably less *MGMT* promoter methylation. By contrast, *MGMT* methylation is common in tumours amplified for *PDGFRA* alone.

Although clearly frequently co-amplified, both *KIT* and *VEGFR2* were less commonly overexpressed at the protein level in response to the increase in DNA copy number, in sharp contrast to *PDGFRA.* Despite this there were significant differences in glioblastoma phenotypes, with isolated KIT receptor-positive tumour cells corresponding with an increased oligodendroglial morphology and *MGMT* promoter methylation, an association not previously reported.

It is intriguing to note that in contrast to tumour expression, receptor positivity in the tumour vascular cells correlated with better clinical outcome for all three RTKs. For PDGFRA and KIT, this amounted to 52% and 26% of cases, respectively. Whilst such an association had not previously been reported for PDGFRA, KIT has been found expressed in tumour endothelial cells in 13/22 glioblastomas, with patients having moderate to strong endothelial cell KIT expression showing a favourable survival than those whose tumour vessels showed little or no expression [32], [33]. More intriguingly, our study reveals a novel subset of cases (8%) to be negative for the otherwise ubiquitous vascular expression of VEGFR2, a cohort which also has a significantly worse outcome. Although there were no apparent biological correlates of these cases, it is an intriguing possibility that we may have identified a novel endothelial-marker negative poor prognosis subgroup of glioblastoma.

## Supporting Information

Table S1 **Full clinicopathological and molecular data for the sample set included in this study.**
(TXT)
